# Effects of Respiratory Muscle Training Pre- and Post-Cardiac Surgery in Adults: A Scoping Review

**DOI:** 10.3390/jcdd11110351

**Published:** 2024-11-02

**Authors:** Giulia Starko, Daniel Müller, Antoine Lipka, Patrick Feiereisen, Camilo Corbellini, Raphael Martins de Abreu

**Affiliations:** 1 Department of Health, LUNEX University of Applied Sciences, 4671 Differdange, Luxembourg; starko.giulia@stud.lunex.lu (G.S.); lipka.antoine@stud.lunex.lu (A.L.); ccorbellini@lunex.lu (C.C.); 2 Department of Physiotherapy, Centre Hospitalier de Luxembourg, 1210 Luxembourg, Luxembourg; mueller.daniel@chl.lu (D.M.); feiereisen.patrick@chl.lu (P.F.); 3 Luxembourg Health & Sport Sciences Research Institute A.s.b.l., 4671 Differdange, Luxembourg

**Keywords:** cardiac therapy, respiratory muscle training, cardiology, cardiac prehabilitation, complications

## Abstract

Background: Coronary artery bypass grafts (CABGs) and cardiac valve replacement surgeries (CVRSs) are common lifesaving cardiac surgeries. They are linked to an increased risk of postoperative pulmonary complications (PPCs). This review scopes the effects of inspiratory muscle training (IMT) on adult patients, considering mainly exercise capacity, lung function, and the occurrence of PPCs. Methods: This scoping review was built using the Preferred Reporting Items for Systematic Reviews and Meta-Analyses extension for Scoping Reviews (PRISMA-ScR). Four databases were searched in May 2024. Three reviewers independently screened the articles. The data were extracted and summarised in text and tables. Results: Five studies were included in the final analysis, where IMT was compared to sham or placebo IMT, and some studies added an exercise program to both groups. PeakVO_2_, the six-minute walking test (6MWT), maximal inspiratory pressure (MIP), quality of life (QoL), PPCs, and spirometry outcomes showed significant improvements between the intervention group (IG) and control group (CG) and intragroup over time. Conclusions: IMT can be a non-conventional training method to prevent respiratory muscle weakness. It can be applied in pre- or post-surgical contexts, potentially affecting exercise capacity and quality of life in adult patients undergoing cardiac surgery.

## 1. Introduction

Coronary artery bypass grafts (CABGs) and cardiac valve replacement surgeries (CVRSs), despite lifesaving, are related to a higher risk of postoperative pulmonary complications (PPCs). The prevalence of PPCs between 5% and 90% has been reported in the literature. PPCs commonly observed include pneumonia, bronchospasms, and respiratory failure [1]. Respiratory muscle weakness at the time of extubation is linked to extubation failure, correlated with increased hospital mortality, and is an independent predictor of one-year mortality [2]. These physiological limitations tend to delay recovery and increase ventilation time, mortality, and morbidity rate post-cardiac surgery [3]. Consequently, addressing the respiratory function pre- and postoperatively is critical to enhance patient outcomes.

A non-invasive therapeutic method to increase respiratory muscle function is respiratory muscle training (RMT). RMT involves exercises and techniques to improve respiratory muscles’ strength and endurance, including the diaphragm and intercostal muscles [4]. Most RMT devices are handheld and function either through a spring-loaded valve that activates when sufficient inspiratory/expiratory pressure is reached or by providing adjustable resistance to inhalation or exhalation airflow. Therefore, the diaphragm and accessory muscles are activated to inhale against resistance repeatedly [5]. Various RMT protocols improved respiratory endurance and strength when applied pre- and post-surgery [1,6]. A previous study indicated that enhancements in respiratory muscle function may not directly translate into clinical practice changes by healthcare professionals since the associated improvements in respiratory muscle function and patient-centred outcomes warrant further investigation [7]. Therefore, this study aimed to evaluate the application of RMT, focusing on respiratory muscle function and its potential impact on recovery, exercise capacity, and reduction in length hospital of stay, which might be improved after RMT [3]. Since RMT is a non-invasive therapy and yields benefits in the recovery of the operated patients, the interest in its perioperative and post-care use is growing.

Many studies have explored the effects of RMT in different contexts. However, there is still no consensus in terms of the dosage of training (i.e., frequency, intensity, type, and time), which makes applying this type of training challenging in clinical practice before and after cardiac surgery [4]. Therefore, this scoping review aimed to explore and provide a comprehensive insight into the effects of RMT pre- and post-surgery in cardiac surgeries. Literature and research gaps will be identified, providing ideas for additional studies on including standardised RMT protocols in perioperative conditions to improve outcomes in patients undergoing cardiac surgery.

## 2. Materials and Methods

### 2.1. Search Strategy

The protocol of this review was registered in the PROSPERO (International Prospective Register of Systematic Reviews) database under the registration number CRD42024557593. The scoping review was conducted following the PRISMA-ScR methodological recommendations [8]. The search strategy was carried out on the 2nd of May 2024 in the following electronic databases: PubMed, Scopus, EMBASE, and EBSCO. The following search strategy was used in each electronic database: “(preoperative rehabilitation OR postoperative rehabilitation) AND (cardiac surgery OR thoracic surgery) AND (breathing exercises OR inspiratory muscle training OR expiratory muscle training OR respiratory muscle training) AND (respiratory muscle strength OR exercise capacity OR functional capacity OR hospital stay)”.

### 2.2. Eligibility Criteria

Initially, all articles were screened considering the following eligibility and inclusion criteria: Inclusion criteria were as follows: (1) adult patients (>18 years), patients undergoing a cardiac surgery of any type, submitted to the perioperative RMT protocol based on resistive load devices compared to sham intervention; (2) randomised control trials, cohort studies, and case-control studies; (3) publications in the English language, full-texts available; (4) study endpoints were either exercise capacity, respiratory muscle function, and length hospital of stay. Considering the number of studies included, we also chose to explore the secondary outcomes, such as lung function, quality of life, and the occurrence of PPCs. Exclusion criteria included any study not meeting the criteria mentioned above.

### 2.3. Study Selection and Data Extraction

Three independent, blinded reviewers conducted the literature screening and selection using a specialised electronic tool (Rayyan.ai). Initially, two reviewers (G.S. and D.M.) selected the articles by title and abstract according to the eligibility criteria. The same two reviewers assessed the full texts of initially selected articles to include the articles in this review. The third independent reviewer resolved any discrepancies or conflicts between the two independent reviewers (A.L.).

The data and results were extracted manually and reported in tables and text. The data extraction included authors, year, country, study design, group size, age, gender, clinical condition, type of surgery, device of RMT training, frequency of RMT sessions, duration of intervention, RMT session duration, RMT intensity, respiratory frequency, control group, primary and secondary outcome measures, and outcome results.

## 3. Results

### 3.1. Study and Participant Characteristics

**Figure 1:**
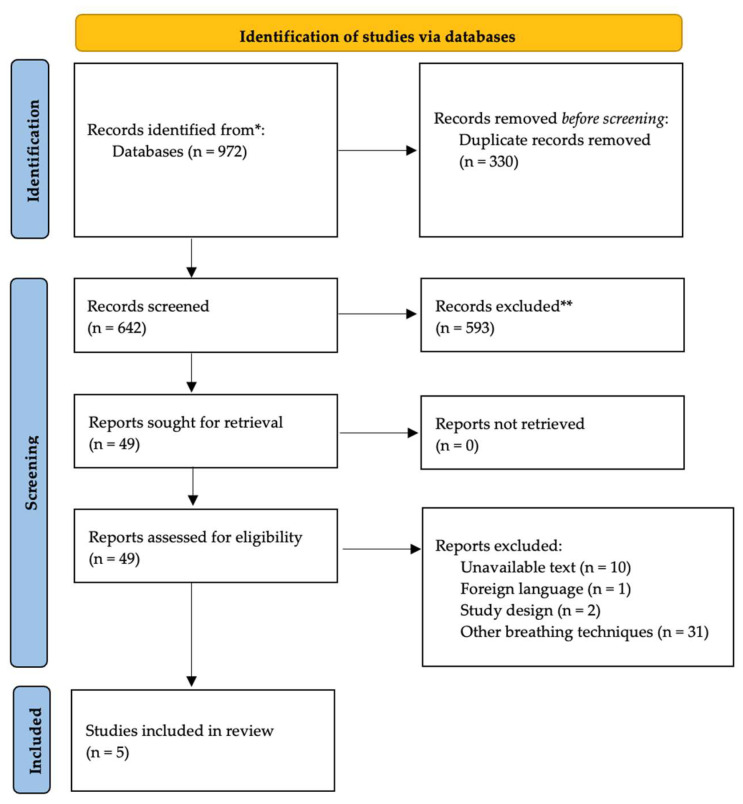
Process flow of the literature search. * PubMed, Scopus, EMBASE, and EBSCO. ** Excluded by automation tools and revised by the reviewers.

In total, 972 articles were identified through our search strategy. After removing duplicates, out of 642 articles screened by title and abstract, only 49 were advanced to full-text screening due to the specific nature of the intervention (i.e., respiratory resistive load training) and the target population (pre- and/or post-cardiac surgery). The total selection process is presented in Figure 1. Finally, five papers were included in this scoping review following the full-text assessment.

The study and patient characteristics of four randomised control trials (RCTs) and one pilot study are presented in Table S1. The sample size varied from 24 to 197, divided equally into two groups. Regarding gender, the ratio of males and females is mostly unequal. All the participants underwent a CABG or CVRS. The clinical condition before surgery was not defined in four papers.

### 3.2. Interventions

The intervention protocols are charted in Table S2. All studies performed either exclusively inspiratory muscle training (IMT) (against threshold) or an IMT in combination with traditional exercise programs, such as endurance and resistance training or breathing exercises. The control group used a sham device in four studies, and only one study used a sham device without resistance. Two studies implemented sham training preoperatively, while three of the included studies conducted it postoperatively.

### 3.3. Outcomes

All papers assessed lung function and exercise capacity. Some evaluated the length of stay, quality of life, and occurrence of PPCs. Table S3 shows the main results of RMT in the primary and secondary outcomes of the selected studies.

#### 3.3.1. Exercise Capacity

Comparing pre- and post-surgery, the intervention group (IG) increased the peakVO_2_ by 22.5% and the control group (CG) by 16.7%, with a significant intragroup difference for both (*p* < 0.001) and a significant intergroup difference (*p* = 0.042) [9]. Another trial showed the IG had a lower improvement than the CG in the follow-up period (*p* = 0.046) [4]. Moreover, three studies assessed exercise capacity using the six-minute walking distance (6MWD). One study showed an increase of 30.30% for the IG and 13.9% for the CG when comparing pre- and post-surgery (*p* < 0.001), showing a significant difference (*p* < 0.001) between both groups [9]. Another study showed a decrease in mean walking distance by 9.7 m in the CG while the IG significantly increased mean walking distance by 35.8 m, with a significant between-group difference (*p* = 0.019) [3]. A third trial revealed that the IG decreased by 16,63 m and the CG by 50.14 m in mean distance, comparing baseline to discharge values, with a significant intergroup difference (*p* = 0.034) [4]. The IG generally decreased the mean 6MWD by 4.9% and the CG by 13.3% [4]. The CG showed a significant gradual improvement over time, but the IG had a greater and more rapid improvement, with an intergroup mean difference of 41.51 m (*p* = 0.041) at one month [4]. In the arm-curl test (ACT) and sit-to-stand (STS) test, there was no difference between both groups, only in the IG over time (*p* = 0.038 and *p* < 0.05, respectively) [4].

#### 3.3.2. Respiratory Muscle Strength and Lung Function

Inspiratory muscle strength (IMS), as measured by maximal inspiratory pressure (MIP), increased in the IG by 15.1% and CG by 3.5%, showing a significant intergroup difference (*p* < 0.001) [1]. Another trial found that the IMS in the IG and the CG showed significant intragroup increases in MIP by 33.7% (*p* < 0.001) and 3.5% (*p* = 0.012), respectively, with a significant intergroup difference (*p* < 0.001) [9]. A third study showed a significantly increased MIP only in the CG (*p* < 0.05) when comparing pre- and post-surgery, with no significant difference between both groups [3]. A different study presented significant intragroup improvements in the IG considering MIP across pre-training and pre-surgery values, followed by a decrease post-surgery (88 cmH_2_O, 101.9 cmH_2_O, 90.4 cmH_2_O, respectively). The CG presented no significant change between pre-training and pre-surgery but a significant decrease post-intervention (94.1 mcmH_2_O, 91.2 cmH_2_O, 74.4 cmH_2_O, respectively), with significant intergroup differences (*p* < 0.001) [10]. The last trial displayed significantly improved MIP in both groups, with a mean increase of 6.60 cmH20 in the IG and 2.35 cmH_2_O in the CG post-surgery [4]. The IG showed significant intragroup improvements over time (*p* < 0.001), with a significant intergroup difference observed only one month post-surgery [4].

The inspiratory muscle endurance (IME) is measured by the relation between peak pressure (Pmpeak), which is a critical measure for assessing inspiratory muscle endurance as it reflects the strength and capability of the inspiratory muscles to generate force and sustain respiratory efforts over time, and MIP. The IME increased significantly in the IG from a mean of 76.1% to 87.0% (*p* < 0.001) at end-training, decreasing significantly to 80.4% post-surgery [10]. In the CG, the IME remained the same before training and pre-surgery at 77.8% and 75.6%, respectively, and decreased significantly post-surgery to 51.9%, with a significant intergroup difference (*p* < 0.001) post-surgery [10]. Another trial expressed the IME as sustained maximal inspiratory pressure (SMIP) and a time limit (Tlim) run of 30 min, showing a significant increase in the IG [9].

Three studies evaluated spirometry outcomes. In the first, forced expiratory volume in one second predicted (FEV1pred), forced vital capacity predicted (FVC pred), and maximal ventilatory volume predicted (MVVpred) increased significantly in the IG, with significant intergroup differences (*p* < 0.05) [1]. Comparing pre-training and post-intervention, another trial found no changes in either group [10]. Post-surgery, only the CG showed a significant mean decrease in FEV1pred and FVCpred, from 90.0% to 80.0% (*p* < 0.005) and 90.0% to 81.0% (*p* < 0.001), respectively [10]. In the last study, FEV1 and FVC improvements were greater in the IG than in the CG at discharge, one month, and three months, and the inverse for FEV1/FVC [4]. Only the FEV1 mean difference at one month showed a significant intergroup difference (*p* = 0.034) [4].

#### 3.3.3. Occurrence of PPCs

PPCs can be graded into four categories as follows: grade 1, dry cough, micro atelectasis, and dyspnoea, not due to other documented causes; grade 2, productive cough, bronchospasms, hypoxemia, atelectasis, hypercarbia, and an adverse reaction to pulmonary medication; grade 3, pleural effusion, suspected and proved pneumonia, pneumothorax, and the need for reintubation; and grade 4, ventilation failure [11]. PPCs were compared in two studies. The CG developed in 27.3% of the patients’ PPCs of grade 2 or higher compared to IG with only 10.2% of cases [1]. The between-group difference was significant (*p* = 0.002), and pneumonia occurrence showed no significant occurrence between both groups [1]. Undefined postoperative complications were reported in 10 and 20 cases, comparing the IG and CG, respectively, with a statistically significant intergroup difference (*p* = 0.028) [4].

#### 3.3.4. Length of Stay (LOS)

Two studies reported LOS as an outcome measure. In one of them, the CG had a mean stay of 9.38 days and the IG of 7.51 days (*p* = 0.039) [4]. The other stated an LOS of 11 days in the IG compared to 12.5 days for the CG (*p* = 0.016) [1].

#### 3.3.5. Quality of Life (QoL)

Quality of life was studied in two trials. The Minnesota living with heart failure questionnaire (MLHFQ) score was reduced by 60.5% in the IG (*p* < 0.001) compared to a decrease of 34.5% in the CG (*p* < 0.001), both being significant over time and between groups (*p* < 0.001) [9]. Considering the short-form 36-item questionnaire (SF-36), the CG showed a significant increase in the physical aspect, pain, vitality, and mental health (all *p* < 0.05) comparing pre- to post-intervention [3]. The IG showed a significant increase in physical aspect, vitality, and mental health (all *p* < 0.05) [9]. No significant difference between both groups was identified [3].

## 4. Discussion

### 4.1. Summary of Evidence

This scoping review studied the effects of RMT before and after cardiac surgery on different outcomes in adult patients undergoing CABGs or CVRSs. Of the 972 initially identified studies, only four RCTs and one pilot study met the inclusion criteria. The sample sizes varied from 24 to 197 participants, predominantly male and at a mean age of 60 years. IMT showed important results for respiratory muscles, exercise capacity, lung function, and the occurrence of PPCs, the LOS, and the QoL in patients pre- and post-surgery. Most protocols were performed 1–2 times per day, 2–7 times per week for approximately 20 to 30 min using an intensity of 30% MIP, then adjusting to higher levels.

Since the objective was to evaluate the effects of isolated RMT, similar studies [12,13] that used alternative breathing techniques or IMT methods (e.g., incentive spirometry, forced expiration technique, cycles of active breathing, etc.) were excluded. Compared to a recent review [13], we included HRVS patients [1,3,4] and CABG patients. This strategy allowed us to observe how IMT affects a larger field of cardiac rehabilitation. By considering sham or placebo treatments in the CG, we could determine that most of the CG showed no significant improvements compared to the IG.

### 4.2. Impact of IMT on Exercise Capacity

Improvements in exercise capacity, measured by VO_2_, were observed in all included studies, especially in the IG [1,4,9]. The 6MWD also showed greater improvements in the IG across various trials; some reported significant gains in mean distance post-surgery or after training. Compared to the CG, the 6MWD in the CG improved slightly or even decreased. IMT mostly led to improved exercise capacity pre- and postoperatively, despite some variability in outcomes across the studies.

The increased respiratory muscle function (i.e., strength and endurance) after IMT has been related to decreased work in breathing, leading to less respiratory fatigue during exercise. One of the main physiological mechanisms involved in the improved exercise capacity post-training is the delay of the respiratory metaboreflex (MBR) [14].

During physical activity, this reflex is initiated by an accumulation of metabolic byproducts in muscles. It is hyperactivated in patients with cardiovascular diseases and is a risk factor, as during physical activity, the blood flow is redirected from peripheral muscles to the respiratory muscles, as these become easily fatigued. Consequently, the decreased perfusion leads to exercise interruption. Improving the respiratory muscles’ function increases their efficiency for longer periods without triggering the MBR. Consequently, peripheral muscle perfusion is maintained for longer periods, thus increasing exercise tolerance. A delayed onset of MBR due to IMT may explain improvements in postoperative exercise capacity, as shown by increased VO_2_max and 6MWD.

### 4.3. Respiratory Muscle Strength and Pulmonary Function

All included studies assessed IMS by focusing on outcome measures such as MIP, while lung function was analysed using the FEV1 and FVC variables [1,3,4,9,10]. Most of the papers reported a significant IMS improvement in the IG, with intergroup differences showing important effects of IMT on improving lung function and IME [9,10]. The assessed spirometry outcomes demonstrated a generally improved IG post-surgery, but not all measures attained statistical significance between both groups.

IMT can be considered resistance training that targets the respiratory muscles, particularly the diaphragm and intercostal muscles. Strength gains increase force-generating and endurance capacities through repeated resistive exercise, leading to hypertrophy of these muscles [15]. Hypertrophy is translated into stronger muscles, generating greater inspiratory pressures. At the same time, increased IME can be explained by increased mitochondrial density, enabling the respiratory muscles to produce more energy and delay fatigue [16].

Consequently, enhanced contractile strength and fibre hypertrophy after IMT can improve the diaphragm-shortening velocity. These adaptations lead to more efficient respiratory mechanics and ultimately enhance lung function, as evidenced by the increased inspiratory capacity (IC) and vital capacity (VC) observed after training [17]. The possibility of deeper inspirations increases the volume of air uptake into the lungs, improving lung expansion and pulmonary function (i.e., forced vital capacity (FVC)). The stronger the respiratory muscles, the greater the negative pressure that can be created, creating more efficient air uptake. However, there are controversies in the literature regarding improving lung functions. Some studies show IMT is not significantly efficient when comparing pre- and postoperative assessments or pre-training and post-training before surgery [10].

### 4.4. LOS and PPCs

Two studies assessed PPCs, finding significantly lower numbers of complications in the IG. One study reported 10.2% occurrence in the IG compared to 27.3% in the CG [1]. The results suggest that IMT reduces the incidence of PPCs, a critical outcome in post-surgery recovery. Two studies evaluated the LOS. Patients in the IG had a significantly shorter LOS than the CG [1,4]. One trial reported a mean stay of 7.51 and 9.38 days for the IG and CG, respectively [1]. A shorter LOS may indicate faster recovery and lower healthcare costs [1,3].

A stronger diaphragm and accessory muscles are crucial for effective breathing, especially after cardiac surgery. IMT applied pre-surgically allows the maintenance of effective ventilation postoperatively [18]. As the lungs can better inflate, the risk of atelectasis decreases. Improved cough effectiveness facilitates clearing lung secretions, preventing pneumonia [1]. A low risk and reduced number of PPCs correlate with a reduced LOS. The weaning from mechanical ventilation postoperatively is faster when the inspiratory muscles are effective. Preventing the occurrence of PPCs decreases mortality and morbidity rates [10]. A shorter LOS implies lower costs that benefit the healthcare system. Avoiding PPCs leads to a faster recovery and return to normal life, resulting in a better QoL.

### 4.5. QoL

The QoL was assessed in two studies using the MLFHQ and the SF-36. The CG showed improvement in the MLFHQ scores (34.5% reduction) over time, but the IG had more significant reductions of 60.5% [9]. While some domains of SF-36 displayed improvements for both groups, there were no significant intergroup differences in QoL improvements [3]. This indicates that IMT may enhance physical recovery, and both groups’ effects on the QoL may be comparable [9]. The absence of significant differences in quality of life between the control and intervention groups, as measured by the SF-36, may reflect the subjective nature of quality-of-life assessments, where individual perceptions and experiences can vary widely [19].

### 4.6. Limitations

This scoping review assessed the effects of isolated RMT (expiratory and inspiratory) pre- and postoperatively. A minor number of five studies could be identified through the literature search. No trials were performed for expiratory muscle training in the included ones. The five included studies show considerable variability in intervention protocols and outcome measures. Three studies focused only on IMT, and the other two on IMT combined with exercises. The missing consistency impedes drawing clear conclusions about the most effective approach to RMT in cardiac surgery.

Since the sample sizes varied from 24 to 197 participants, the statistical power to identify significant differences is limited, predominantly in secondary outcome measures like QoL or PPCs. The participants were predominately male, giving an unbalanced gender ratio. The generalisation of the results may be limited as male and female patients could have different reactions to cardiac surgery and rehabilitation. Since the preoperative clinical conditions are unknown, it is difficult to determine the baseline health status, which may impact the rehabilitation process. This also leads to unknown results if a patient with a more severe condition benefits more or less from IMT.

Although telerehabilitation is currently being used with higher frequency, no study regarding the search and screening strategy was included. Given the characteristics of IMT, which is less dynamic than other training modalities and does not necessarily require supervision, it could serve as a valuable component in cardiac telerehabilitation (CTR). Over time, many factors have developed in this type of rehabilitation, which allow easier geographical accessibility, increased patient participation, and better cost and time effectiveness [20]. Cardiovascular and QoL outcomes were similar or improved significantly in the IG receiving TR compared to the CG supervised by a medical professional [20]. A reduced risk of postoperative complications was shown in elective cardiac surgery due to telerehabilitation.

### 4.7. Future Perspectives

The included trials had various outcome measures, but the assessment tools differed between the studies—for example, exercise capacity measured by peak VO_2_ or 6MWT. This complicates the comparison between the studies and the generalisation of the results. The number of outcome measures was limited and included more physical aspects. Adverse events, well-being, and patient satisfaction were not assessed, which are also crucial to recovery. Moreover, the follow-up period was short-term, up to three months post-surgery. The long-term effects of IMT remain unclear. Future studies should assess the long-term effects of IMT in different groups (based on clinical condition and age) of patients. Using the same outcome measures for easier comparison and generalisation of the results would be important. The cognitive, mental, and well-being domains should also be assessed in future studies. Furthermore, future research should investigate the potential effects of expiratory muscle training protocols, which have not been extensively explored when compared to IMT.

## 5. Conclusions

This scoping review underlines the potential benefits of IMT for adults undergoing cardiac surgery. Findings show that IMT can significantly improve exercise capacity, lung function, and IMS in pre- and post-surgery settings. Although the protocols were variable, the application of IMT tends to reduce the PPCs, shortening the LOS and possibly impacting healthcare costs. The quality of life in the IG and CG improved significantly for both intergroups.

However, the variability in participant characteristics (i.e., gender, unclear preoperative clinical conditions) and intervention protocols limits the generalisation of these findings. Future research should focus on larger sample sizes. Moreover, a long-term follow-up should clarify if IMT provides durable improvements in postoperative recovery, as well as the recurrence of a new cardiac event and long-term levels of physical capacity.

## Data Availability

The authors confirm that the data supporting the findings of this study are available within the article.

## Supplementary Materials

The following supporting information can be downloaded at: https://www.mdpi.com/article/10.3390/jcdd11110351/s1, Table S1. Study Characteristics. Table S2. Characteristics of intervention. Table S3. Outcomes and Results.
