# Supplementary material for: Effects of Respiratory Muscle Training Pre- and Post-Cardiac Surgery in Adults: A Scoping Review
Source: J Cardiovasc Dev Dis. 2024 Nov 2;11(11):351. doi: 10.3390/jcdd11110351 (PMC11594498; doi:10.3390/jcdd11110351)
Supplement: Supplementary file 1 [file jcdd-11-00351-s001.zip › jcdd-3261495-supplementary.pdf]

Table S1. Study Characteristics.

First author, year	Country	Study design	Groups (n)	Age (years ± SD)	Gender (M/F)	Clinical condition	Type of surgery
Chen, et al., 2019	China	Prospective Single-blind randomized controlled pilot study.	IG n=98<br>CG n=99	CG: 61.68+-8.12<br>IG: 61.68+-7.73	IG: 73 M / 25 F<br>CG: 68 M / 31 F	n.r.	CBAG and/or HVRS
Dos Santos, et al., 2019	Brazil	RCT double-blind, single-center	IG (n =12)<br>CG (n=12)	IG: 55.0+-7.0<br>CG: 56.6+-5.5	IG: 8 M / 4 F<br>CG: 9 M / 3 F	n.r.	CBAG
Cargnin, et al., 2019	Portugal	double-blind, RCT	IG (n=13)<br>CG (n=12)	IG: 62.8+-8.8<br>CG: 60.1+-12.5	IG: 9 M / 4 F<br>CG: 6 M / 6 F	n.r.	HVRS
Weiner, et al., 1998	Israel	RCT	IG (n=42)<br>CG (n=42)	IG: 59.2+-3.8<br>CG: 63.8+-3.1	58 M / 26 F	n.r.	CBAG
Xu, et al., 2023	China	RCT single-centre clinical, double-blinded	IG (n=48)<br>CG (n=48)	IG: 72.01+-5.56<br>CG: 72.45+-7.68	IG: 25 M / 23 F<br>CG: 29 M / 19 F	AS	TAVR

Abbreviations: randomized control trial (RCT); intervention group (IG); number of participants (n); control group (CG); standard deviation (SD); male (M); female (F); not reported (n.r.); aortic stenosis (AS), cardiac bypass artery grafting (CBAG); heart valve replacement surgery (HVRS); Transcatheter aortic valve replacement (TAVR)

Table S2. Characteristics of intervention

First author, year	Device	Frequency (times/week)	Duration of intervention(weeks)	Session duration (min)	Intensity	Respiratory Frequency (bpm)	Control group
Chen, et al., 2019	Threshold IMT device (HS730-010; Philips Respironics, Pittsburgh, PA, USA)	2x/day	5 days pre-op	20min	adjusted to maintain 30% of MIP	5 breaths and rest 5-10sec during 20min	same protocol, constant intensity at min. load of the device (9cmH2O)
Dos Santos, et al., 2019	IMT: POWERbreathe® Medic Plus (POWERbreathe International Ltd., England, UK)<br><br>CT : motorized treadmill (ATL 10200, Inbramed, Porto Alegre, RS, Brazil)	2x/week	12 weeks post-op	IMT : n.a.<br><br>CT : 60min	IMT: week 1-2: 50% MIP<br><br>weekly increase of 5% MIP<br><br>week 9-12: maintance 80% MIP<br><br>CT : 30min treadmill with HR obtained of VT1 at baseline CPET, 4-6 RPE (mBORG),<br><br>3x10 reps/muscle group + 10s rest, at 50% 1RM	IMT : 5x 10 reps	IMT: same protocol. min. load of device (9cmH2O)<br><br>CT: same protocol
Cargnin, et al., 2019	POWERBreathe Kinetic KH1 (POWERBreathe International)	2x/day + 7days/week	4 weeks post-op	n.a.	weekly adjusted to 30% of MIP	30 ventilatory cycles	Same protocol with min. load of device

Table S2. Continued

First author, year	Device	Frequency (times/week)	Duration of intervention(weeks)	Session duration (min)	Intensity	Respiratory Frequency (bpm)	Control group
Weiner, et al., 1998	Threshold Inspiratory Muscle Trainer, Health- scan, NJ, USA	6x/week	2-4 weeks pre-op (depending on surgery date)	0,5h	15% of their PImax or for 1 week increased incrementally: 5% each session up to 60%	n.a.	Same protocol, without resistance
Xu, et al., 2023	respiratory training device (Blue WhaleTM, Xeek, Xiamen, China) with re-<br>sistance load	Once/day, 3-5 days/week	from 2nd day post-op, during hospital stay	Exrcises: 30min<br><br>IMT: 20min	Exercises: Transfers, walking<br><br>IMT:<br>Warm-up: 5 min of breathing control training, chest expansion, stretching<br><br>Main:<br>10 min initially 30%MIP, gradually increasing to min. 60%<br><br>Cool down: 5 min <30% of MIP	n.a.	Same protocol, sham device with load <1 cmH2O

Abbreviations: preopertive(pre-op); postoperative (post-op); inspiratory muscle training (IMT); minimal (min); combined training (CT); maximal inspiratory pressure (MIP); HR (heart rate); ventilatory threshold 1 (VT1); cardiopulmonary exercise test (CPET), rate of perceived exertion (RPE), modified BORG (mBORG); one repetition maximal (1RM)

Table S3. Outcomes and Results.

First author, year	Primary outcomes measures	Secondary outcome measures	Outcome results
Chen, et al., 2019	occurrence of PPC	Pimax, FEV1pred, FVCpred, MVVpred, LOS	PCC: IG↓ CG↓ (intergroup sign ≠)<br>Pneumonia: IC + CG (intergroup no sign ≠)<br>Pimax IG ↑ CG ↑(intergroup sign ≠)<br>FEV1 %predicted IG ↑ CG ↑ (intergroup sign ≠)<br>FVC %predicted IG ↑ CG ↑ (intergroup sign ≠)<br>MVV %predicted IG ↑ CG ↑(intergroup sign ≠)<br>LOS: IG↓ CG↓ (intergroup sign ≠)
Dos Santos, et al., 2019	peak VO2	MIP, 6MWT, MLHFQ, SMIP, Tlim	peakVO2: IG ↑ CG↑ (inter-, intragroup sign ≠)<br>6MWT: IG ↑ CG↑ (inter-, intragroup sign ≠)<br>MIP: IG ↑ CG↑ (intergroup sign ≠)<br>MLHFQ: IG ↓ CG↓ (inter-, intragroupsign ≠)<br>SMIP: IG↑ CG↑<br>Tlim: IG↑ (sign ≠) CG↑
Cargnin, et al., 2019	MIP	6MWT, SF-36, FEV1, FVC, FEV1/FVC	MIP: IG ↓ then ↑ CG ↓ then ↑ (intragroup sign ≠, post-op intergroup sign ≠)<br>6MWT: IG ↑ (sign ≠) CG↓ (intergroup sign ≠)<br>FEV1, FVC, FEV1/FVC:<br>Pre-op + post-op IG ↑ (no sign ≠) CG ↑ (sign ≠), (no intergroup sign ≠)<br>SF-36: IG ↑ CG ↑ (intergroup sign ≠, no sign ≠ intragroup)
Weiner, et al., 1998	PImax<br>Pmpeak/PImax	FEV1, FVC	PImax:<br>IG pre-op↑ (sign ≠), post-op↓<br>CG pre-op ←→, post-op ↓ (sign ≠)<br>Pmpeak/Pipeak:<br>IG pre-op↑ (sign ≠), post-op↓<br>CG pre-op ←→, post-op ↓ (sign ≠)<br>(intergroup sign≠ post-op)<br>FEV1 + FVC:<br>IG ←→<br>CG pre-op ←→, post-op↓ (sign ≠)

Table S3. Continued

First author, year	Primary outcomes measures	Secondary outcome measures	Outcome results
Xu, et al., 2023	6MWT	FVC, FEV1, FEV1/FVC, MIP, LOS, peakVO2, UPC, STS, ACT, BI	6MWT:<br>baseline to discharge: IG↓ CG↓ (intergroup sign ≠)<br>discharge to 1 month: IG↑ CG↑ (intergroup sign ≠)<br>MIP: IG↑ (sign ≠) CG↑<br>FVC, FEV1, FEV1/FVC: IG + CG (no sign intergroup ≠)<br>peakVO2: IG↑ CG↑↑ (intergroup sign ≠)<br>STS + ACT: IG↑ CG←→ (no intergroup ≠, only over time intragroup sign ≠ in IG)<br>BI: IG↑ CG ↑ (intergroup sign ≠ at 1 month)<br>LOS: IG↓ CG↓ (intergroup sign ≠)<br>UPC: IG↑ CG↑↑ (intergroup sign ≠)

Abbreviations: improvement/increase (↑); worsening/decrease (↓); unchanged (←→); postoperative pulmonary complications (PPC); maximal inspiratory mouth pressure (Pimax); forced expiratory volume in one second predicted (FEV1pred); forced vital capacity predicted (FVCpred); maximal ventilatory volume predicted (MVVpred); length of stay (LOS); six minute walking test (6MWT); Minnesota living with heart failure questionnaire (MLHFQ); sustained maximal inspiratory pressure (SMIP); time limit run of 30min (Tlim); short-form 36-item questionnaire (SF-36); forced expiratory volume in one second predicted (FEV1); forced vital capacity (FVC); peak pressure (Pmpeak); undefined postoperative complications (UPC); sit to stand test (STS); arm-curl test (ACT); Barthel index (BI)
